# Mysterious cases of acute hepatitis in children: is adenovirus still a lead suspect?

**DOI:** 10.1080/22221751.2022.2095933

**Published:** 2022-07-17

**Authors:** Adriana E. Kajon, Kirsten St. George

**Affiliations:** a Infectious Disease Program, Lovelace Biomedical Research Institute, Albuquerque, NM, USA; b New York State Department of Health, Wadsworth Center, Albany, NY, USA; c Department of Biomedical Science, University at Albany, NY, USA

**Keywords:** Adenovirus, paediatric hepatitis, adenovirus type 41

## Commentary

As this commentary is being written, the number of probable cases of acute hepatitis of unknown origin in children reported worldwide continues to rise, now exceeding 650. Thirty-three countries representing all continents have reported cases, with the UK accounting for the highest number with 222 [1]. Notably, most are sporadic un-linked cases with the majority being under 5 years of age. Thirty-eight children have required liver transplants and nine fatalities have been documented.

While efforts to thoroughly investigate and identify the cause(s) of this mysterious illness are ongoing, health alerts and guidelines for sample collection, testing, and reporting have been released across the globe by public health authorities. So far, no firm explanations for the contemporaneous occurrence of similar cases in geographically distant nations have been found.

The topic was discussed in a late breaker session by co-author AEK on May 3 at the Clinical Virology Symposium, organized by the American Society for Microbiology, and since then several editorials, commentaries, and letters to editors have been contributed to various journals, and social media blogs, raising awareness [2,3].

Under the case definition released by the Centers for Disease Control and Prevention in the USA (CDC), “Children <10 years of age with elevated aspartate aminotransferase (AST) or alanine aminotransferase (ALT) (>500 U/L) who have an unknown aetiology for their hepatitis,” the number of persons under investigation in the United States alone now involves 38 states since 1 October 2021, and was collectively reported by the CDC to be 246 as of 1st June [4].

The intriguing detection of adenovirus by qPCR in a considerable number of the cases investigated so far, and in particular of enteric type HAdV-F41 [5] has attracted a lot of attention and debate in the clinical virology and public health communities. Adenoviruses are not typically hepatotropic and are rarely associated with acute hepatitis in immunocompetent individuals. Reviews of paediatric adenovirus hepatitis report the disease to occur most commonly in liver and stem cell transplant recipients, patients receiving chemotherapy for solid malignancies, and those with severe combined immunodeficiency, and most commonly to be caused by species C adenoviruses, especially type 5 [6]. Interestingly, adenovirus and other respiratory infections have been documented to cause a transient “reactive hepatitis” in children [7].

Both of the human species F adenoviruses, types 41 and 40, are causative agents of paediatric gastroenteritis [8] with type 41 being more prevalent than type 40. However, both types are seldom detected in other contexts of paediatric disease [9]. Significant intratypic genetic variability has been demonstrated for HAdV-F41 with genomic sequencing and phylogenetic analysis, and two major clades have been described [10]. The extremely low viral loads in clinical specimens received from paediatric hepatitis cases thus far have caused typing approaches to be limited to PCR amplification of a portion of the hexon gene followed by Sanger sequencing. In the cohort of patients from Alabama, USA, three different hexon variants were identified among the cases where typing was possible, supporting the conclusion that they were not epidemiologically related (St George, personal communication). Importantly, typing data available for four of the European cases testing positive for adenovirus showed type F41 in two cases, type F40 in one case and a type “other” in one case, further evidence of the absence of relatedness between cases [11]. These data suggest that an ongoing enteric adenoviral infection could potentially have a pathogenic effect on the liver via the “gut-liver” axis, which warrants further investigation. The possibility that mutations in the genome of recently circulating strains of HAdV-F41 have resulted in viruses with altered tropism or pathogenicity cannot be ruled out at this point but whole genome sequence data will be required to evaluate this. To the best of our knowledge, no full genome sequence data are currently available for the adenovirus strains detected in any of the recent cases of paediatric hepatitis.

Although informative, the detection of concurrent viral infections is not sufficient to prove causality, and an expanded variety of clinical specimens needs to be collected from as many affected individuals as possible to add critical pieces to this difficult puzzle, in order to better investigate and understand the involvement of various organs and anatomical compartments.

With COVID-19 mitigation practices in recent years, the prevalence of many respiratory and other infectious diseases decreased significantly. With the majority now returning to pre-pandemic levels we postulate that the recently observed hepatitis cases may be due to adenovirus, and likely other co-infections, triggering exacerbated inflammatory responses targeting a sensitized liver. The lack of detection of characteristic nuclear inclusion bodies, viral antigen by immunohistochemistry, or nucleic acid by *in situ* hybridization, strongly suggests that adenovirus infection is not directly responsible for the observed liver pathology. As demonstrated for various other non-hepatotropic viruses, systemic infections may cause hepatic injury ranging from mild to severe acute hepatitis and occasionally acute liver failure [12]. The described histopathology in many of the recent cases of acute paediatric hepatitis may be an example of this kind of “collateral damage.”

A history of exposure to SARS-CoV-2 is a strong contender for a pre-existing condition and a common feature of paediatric populations around the world. While SARS-CoV-2 serology investigations in the reported paediatric hepatitis cases are still ongoing, very little is currently known about long COVID in children, especially regarding effects on liver function. However, evidence for the hepatic tropism of SARS-CoV-2 and molecular signature data from the characterization of associated liver injury provides strong incentive to pursue further study in this area [13]. The recently published report on documented long COVID-19 liver manifestations in children [14] adds significant fuel to the ongoing debate. Importantly, an additional mechanism has been proposed, where release of viral proteins across the intestinal epithelium may mediate a superantigen motif in the SARS-CoV-2 spike protein, in adenovirus-sensitized hosts [15] which also warrants further investigation.

Multidisciplinary teams of investigators including immunologists, virologists, pathologists, and epidemiologists must be assembled to assist with the ongoing investigations to provide mechanistic insights on the pathogenesis of this intriguing illness.
